# Alterations to the Gut Microbiota and Their Correlation With Inflammatory Factors in Chronic Kidney Disease

**DOI:** 10.3389/fcimb.2019.00206

**Published:** 2019-06-12

**Authors:** FengXia Li, MeiHong Wang, JunPing Wang, RongShan Li, YaQiong Zhang

**Affiliations:** ^1^Department of Gastroenterology, Shanxi Provincial People's Hospital, Taiyuan, China; ^2^People's Hospital Affiliated to Shanxi Medical University, Taiyuan, China; ^3^Department of Nephrology, Shanxi Provincial People's Hospital, Taiyuan, China

**Keywords:** gut microbiota, chronic kidney disease, inflammatory factors, 16S rDNA deep sequencing, *Akkermansia*

## Abstract

Alterations to the gut microbiota have been previously suggested to be tightly linked to chronic systemic inflammation, which is a major contributing factor to complications and disease progression in chronic kidney disease (CKD). Nevertheless, the effect of gut dysbiosis on the pathogenesis and/or production of inflammatory factors in CKD has not been extensively studied to date. In the present study, we conducted 16S ribosomal DNA pyrosequencing using fecal microbiota samples and analyzed the production of serum inflammatory factors in 50 patients with CKD and 22 healthy control (HC) subjects. The results revealed that compared to the HC subjects, patients with CKD exhibited a significant reduction in the richness and structure of their fecal microbiota. At the phylum level, compared to the HC group, patients with CKD also presented reduced abundance of Actinobacteria but increased abundance of Verrucomicrobia. Moreover, the genera *Lactobacillus, Clostridium IV, Paraprevotella, Clostridium sensu stricto, Desulfovibrio*, and *Alloprevotella* were enriched in the fecal samples of patients with CKD, while *Akkermansia* and *Parasutterella* were enriched in those of the HC subjects. The abundance of *Akkermansia* in the CKD group was significantly lower than that in the HC group (3.08 vs. 0.67%); this decrease in the abundance of *Akkermansia*, an important probiotic, in patients with CKD is a striking discovery as it has not been previously reported. Finally, we analyzed whether these changes to the fecal microbiota correlated with CKD clinical characteristics and/or the production of known inflammatory factors. Altered levels of the microbiota genera *Parasutterella, Lactobacillus, Paraprevotella, Clostridium sensu stricto*, and *Desulfovibrio* were shown to be correlated with CKD disease-severity indicators, including the estimated glomerular filtration rate. Most notably, *Akkermansia* was significantly negatively correlated with the production of interleukin-10. The results of the present study suggest that microbiota dysbiosis may promote chronic systemic inflammation in CKD. Furthermore, they support that modifying the gut microbiota, especially *Akkermansia*, may be a promising potential therapeutic strategy to attenuate the progression of, and/or systemic inflammation in, CKD.

## Introduction

Chronic kidney disease (CKD) is a major public health problem that affects an estimated 350 million people worldwide and results in 0.5–1 million deaths annually. Furthermore, patients with CKD often develop cardiovascular disease (CVD) as a complication of their condition and resultantly experience an extremely high rate of cardiovascular-associated mortality (Webster et al., 2017). Several risk factors have been well-established to contribute to cardiovascular complications in CKD, including obesity, hypertension, diabetes, and dyslipidemia; however, novel risk factors have also been recently identified to promote cardiovascular complications in a large number of patients with CKD, including hyperhomocysteinemia, increased lipoprotein, oxidative stress, and chronic systemic inflammation (Mafra et al., 2014). In particular, a growing body of evidence suggests that chronic systemic inflammation is a major risk factor for CKD-associated CVD pathogenesis and progression (Qian, 2017).

Recent studies indicate that chronic systemic inflammation in CKD is closely linked to the gut microbiota. Consistent with this finding, the balance of gut microbiota is well-established to be beneficial to the host and to play a fundamental role in the metabolism of dietary fibers, carbohydrates, and proteins that are not degraded by human enzymes, as well as in vitamin (e.g., B and K) synthesis, production of short-chain fatty acids (SCFAs) that provide nutrition for intestinal epithelial cells, immune-system stimulation, and maintenance of intestinal epithelium homeostasis (Sonnenburg and Bäckhed, 2016). Conversely, when the gut microbiota becomes dysbiotic, pathogenic bacteria overgrow and secrete increased amounts of bacterial products such as lipopolysaccharides, peptidoglycans, and bacterial DNA and/or outer-membrane proteins into the host circulatory system, thereby causing chronic immune activation. Moreover, these harmful substances destroy intestinal permeability (Anders et al., 2013; Sabatino et al., 2015) and activate the intestinal-mucosa immune system (Hand et al., 2016), thereby promoting the generation of inflammatory factors such as interleukin (IL)- 6, interferon γ (IFN-γ), and tumor necrosis factor α (TNF-α) (Rossi et al., 2014). Persistent immune activation is a major risk factor for both CKD progression and cardiovascular complications (Darisipudi and Knauf, 2016); thus, identifying bacteria associated with chronic systemic inflammation and elucidating the role and mechanisms by which the altered gut microbiota contributes to chronic systematic inflammation are of urgent importance to improve current CKD therapies.

Some previous studies have shown changes to the intestinal microbiota of patients with CKD. For example, Vaziri et al. (2013) observed marked differences in the abundance of nine families of bacteria in patients with end-stage renal disease (ESRD) compared to healthy control (HC) subjects. Nevertheless, little is known regarding gut-microbiota alterations that are associated with chronic systematic inflammation in CKD in Chinese populations. In the current study, we analyzed and compared the gut microbiota in the feces of patients with CKD and HC subjects via 16S rDNA gene sequencing; we further analyzed the relationships between fecal microbiota and the clinical characteristics of and inflammation in patients with CKD. Thus, the present study aimed to elucidate CKD-associated differences in fecal microbial communities as well as associations among gut dysbiosis, clinical characteristics, and chronic systematic inflammation in patients with CKD. We unexpectedly found that the abundance of the genus *Akkermansia* was significantly decreased in CKD patients; this decrease in the abundance of *Akkermansia*, an important probiotic, in patients with CKD is a striking discovery as it has not been previously reported. *Akkermansia* has been shown to have important implications in host physiology, and is particularly effective in increasing both mucus thickness and gut barrier function (Ottman et al., 2017a), and thus, its decrease in CKD patients might have an effect on the disease progression. The generated data provide promising novel therapeutic microbiota targets, including *Akkermansia* to attenuate the progression of, and reduce the risk of cardiovascular complications in, CKD.

## Materials and Methods

### Study Design

The present study enrolled 50 patients with CKD and 22 HC subjects from the Departments of Nephrology and Health Examination of the Shanxi Provincial People's Hospital (Shanxi Medical University, Shanxi, China), respectively, between June 2017 and February 2018. All study subjects provided written informed consent for their participation in the study, the design of which was approved by the Research Ethics Committee of the Shanxi Provincial People's Hospital.

Patients were deemed eligible for this study if they were diagnosed with CKD because they exhibited an effective glomerular filtration rate (eGFR) of <60 ml/min/1.73 m^2^ for a 3-month period, while the HC subjects exhibited no CKD disease symptoms. Subjects (both with and without CKD) were excluded if they exhibited any other serious chronic illnesses (e.g., liver cirrhosis, malignancy, or hematological or autoimmune diseases), acute intercurrent infections, and/or were administered antibiotics, probiotics, or immunosuppressive drugs within 3 months prior to enrollment in the study. Patient clinical data [including weight, height, and body mass index (BMI)] were collected by and obtained from the Department of Clinical Laboratory of Shanxi Provincial People's Hospital. Feces and serum samples from participants were collected, transferred on ice, and stored at −80°C until use.

### Measurement of Serum Levels of Inflammatory Cytokines

Serum samples (10 ml) were collected from subjects at the time of the first fecal-sample collection. Levels of the inflammatory cytokines IL-6, IL-4, and IL-10 were quantitatively detected using a Quantitative Cytokine Quantibody Human Array (RayBiotech, Norcross, GA, USA), according to the manufacturer's instructions. Briefly, antibodies against different cytokines were spotted onto a high-throughput cytokine array, and the resulting green fluorescence (Cy3 channel, 532-nm excitation) was captured using an InnoScan 300 Microarray scanner (Innopsys, Carbonne, France). Images were visualized using Quantitative Cytokine Antibody Array software (RayBiotech), and data were extracted and analyzed using (“GAL”) files and (QAH-TH1-1) software that were provided with the Quantitative Cytokine Quantibody Human Array (RayBiotech).

### DNA Extraction and Amplicon and Sequencing

Microbial DNA was isolated from fecal samples using the QIAamp DNA Stool Mini Kit (Qiagen, Valencia, CA) according to the manufacturer's instructions. The amount of DNA in each fecal sample was quantified using a Thermo NanoDrop 2000 spectrophotometer (Thermo Scientific, MA, USA). DNA integrity and size were confirmed via 1% agarose gel electrophoresis.

DNA sequencing libraries targeting the V3–V4 hypervariable regions of the 16S rRNA gene were prepared by PCR amplification using specific primers supplemented with Illumina sequencing adapters and sample-specific barcodes according to Illumina's instructions (https://support.illumina.com/downloads/16s_metagenomic_sequencing_library_preparation.html). The PCR reactions were performed as follows: 95°C for 3 min, followed by 30 cycles of 98°C for 20 s, 58°C for 15 s, and 72°C for 20 s, with a final extension of 72°C for 5 min. PCR reactions (30 μL) were conducted using 1 μL of appropriate (10 μM) primers (*341F*, 5′-CCTACGGGRSGCAGCAG-3′; *806R*, 5′-GGACTACVVGGGTATCTAATC-3′), 15 μL of 2 × KAPA Library Amplification ReadyMix (Kapa Biosystems, Roche, MA, USA), 50 ng template DNA, and ddH_2_O.

Amplicons were extracted via electrophoresis using 2% agarose gels, purified using the AxyPrep DNA Gel Extraction kit (Axygen Biosciences, Union City, CA, USA) according to the manufacturer's instructions, and quantified using the Qubit®2.0 fluorometer (Invitrogen, USA). After library preparation, the HiSeq platform (Illumina, Inc., CA, USA) was used to sequence 16S tags that comprised 250 bp paired-end reads that overlapped at their 3′ ends (to enable concatenation into original longer tags). DNA extraction, library construction, and sequencing were conducted by the Realbio Genomics Institute (Shanghai, China).

### Bioinformatic and Statistical Analyses

Length and quality of each 16S tag were evaluated after trimming of barcodes and primers. Tags were selected for further analysis if they were 220–500 bp in length, displayed an average Phred score of bases no worse than 20 (Q20) and included no more than three ambiguous bases. The tag copy numbers were then analyzed, and all redundant (repeated) tags were removed. Only tags that were detected with a frequency > 1 were further selected and clustered into Operational Taxonomic Units (OTUs) with 97% similarity using UPARSE software (http://drive5.com/uparse/). Chimeric sequences were identified and removed using Usearch software (ver. 7.0). Each OTU was assigned a Representative OTU tag using RDP Classifier software (http://rdp.cme.msu.edu/) and the RDP database (http://rdp.cme.msu.edu/), with a confidence threshold of 0.8. OTU-profiling tables were constructed, and alpha/beta diversity analyses were conducted using python scripts obtained from Qiime (http://qiime.org/).

Statistical analyses, including independent *t*-, Welch's *t*-, and Mann-Whitney *U*-tests were conducted using SPSS (ver. 21.0, SPSS Inc., Chicago, IL, USA) and R (ver. 3.1.0, the R Project for Statistical Computing) software. Spearman's correlation coefficient was calculated using SPSS Version 16.0 (SPSS Inc., Chicago, IL) and GraphPad Prism 5 (GraphPad Software, Inc.) software and used to assess bivariate relationships between variables. All tests of significance were two-sided, and *p* (or corrected *p*) values < 0.05 were considered to indicate statistical significance.

## Results

### Basic Characteristics of Patients With CKD and HC Subjects

The present prospective cross-sectional study enrolled and conducted anthropometric and biochemical phenotyping for 50 patients with CKD and 22 HC subjects (Table 1). The subjects in the two groups exhibited similar age, gender, and BMI distributions. Various clinical biochemical metabolic markers were compared between individuals with and without CKD to identify CKD-induced changes to renal function and/or chronic inflammation. The results of this analysis showed that the subjects in the CKD group had significantly higher blood urea-nitrogen (BUN) (*P* < 0.0001), serum creatinine (SCr) (*P* < 0.0001), and Cystatin-C (CysC) (*P* < 0.0001) levels than those in the HC group. In addition, the patients with CKD had a markedly lower estimated glomerular filtration rate (eGFR) (*P* < 0.0001) and carbon dioxide combining power (CO_2_CP) (*P* < 0.0001) than those in the HC subjects, with all these markers being indicative of biochemical disturbances associated with renal dysfunction. Moreover, analysis of subject serum samples showed that serum levels of the inflammatory cytokines IL-6, IL-4, and IL-10 were significantly increased in patients with CKD compared with HC subjects (*P* < 0.05).

**Table 1:** Clinical characteristics and inflammatory cytokine levels exhibited by patients with chronic kidney disease (CKD) and healthy control subjects (HC).

**Clinical characteristics**	**CKD group** **(*n* = 50)**	**HC group** **(*n* = 22)**	***P*-value**
Sex (male/female)	27/23	12/10	–
Age (years)	52.40 ± 13.49	50.27 ± 7.77	0.403
BMI (kg/m^2^)	24.05 ± 3.86	24.31 ± 3.19	0.780
eGFR (mL/min/1.73 m^2^)	22.39 ± 15.56	97.06 ± 12.98	<0.001
SCr (mmol/L)	365.36 ± 243.18	70.18 ± 10.57	<0.001
BUN (mmol/L)	16.15 ± 8.29	4.18 ± 1.56	<0.001
CO_2_CP (mmol/L)	20.83 ± 5.15	25.55 ± 1.87	<0.001
CysC (mmol/L)	3.78 ± 1.23	0.96 ± 0.19	<0.001
IL-4 (pg/ml)	1.95 ± 1.89	0.76 ± 0.42	0.001
IL-6 (pg/ml)	30.88 ± 11.44	24.18 ± 5.69	0.004
IL-10 (pg/ml)	1.14 ± 0.98	0.59 ± 0.31	0.014

*Data are shown as the mean ±SD. BMI, body mass index; BUN, blood urea-nitrogen; CO_2_CP, carbon dioxide combining power; CysC, Cystatin C; eGFR, estimated glomerular filtration rate; IL, Interleukin; SCr, serum creatinine*.

### Differences in Fecal Microbiota Diversity Between CKD and HC Subjects

**Figure 1:**
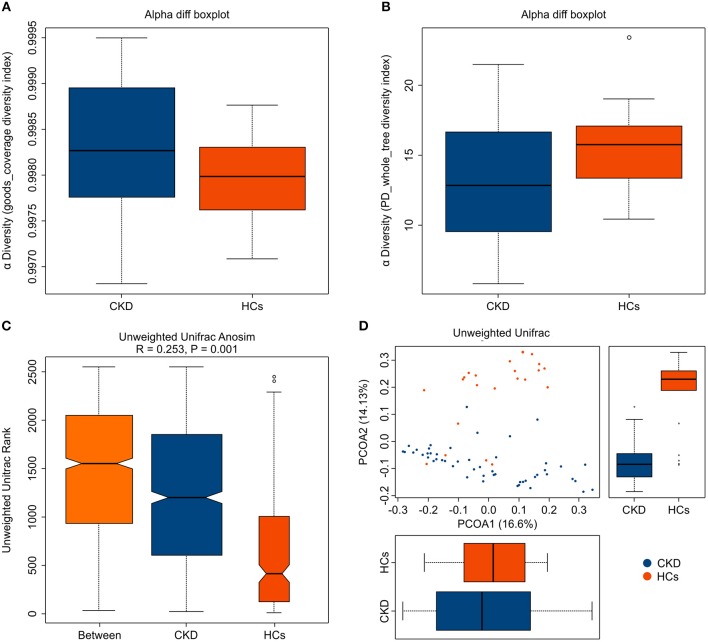
Fecal microbiota alpha- and beta-diversity in patients with chronic kidney disease (CKD) and healthy control (HC) subjects. The depicted box plots show differences in the fecal microbiome diversity indices between the CKD and HC groups, as assessed using **(A)** Goods coverage diversity and **(B)** Phylogenetic Diversity (PD) whole-tree indices. Each box plot represents the median, interquartile range, minimum, and maximum values. **(C,D)** The level of similarity between the fecal microbial communities detected in the CKD (blue) and HC (orange) groups was assessed via **(C)** an unweighted Analysis of similarity (ANOSIMs) and **(D)** a principal coordinates analysis (PCoA; based on the UniFrac distance matrix). Respective ANOSIM R values show community variation between the compared groups, and significant *P*-values are indicated. Each symbol represents a sample.

A total of 2,276,325 high-quality sequences (comprising 88.17% of the total 2,581,727 generated valid reads) were produced from fecal samples collected from the 72 analyzed subjects and were shown to have a median read length of 417 bp (range, 411–425 bp). An average of 35,857 (range, 33,048–38,970) sequences/barcoded samples were recovered for downstream analysis. A total of 12,981 unique sequences were generated from the two subject groups and confirmed to represent all phylotypes. The Good's coverage for the two groups was more than 99.0%, which indicates that sufficient sequencing depth was achieved to accurately represent the CKD-associated fecal microbiota (Figure 1A).

Analysis of the collected fecal samples revealed that the mean community diversity (represented by the phylogenetic diversity (PD) whole-tree index) was significantly reduced in the CKD compared with the HC group (*P* < 0.05) (Figure 1B). Moreover, Shannon and Simpson diversity analyses showed that the alpha diversity was markedly, but not significantly, lower in the fecal microbiota obtained from the patients with CKD than in those from the HC subjects (*P* > 0.05). An analysis of similarity (ANOSIM) and principal coordinates analysis (PCoA; based on unweighted UniFrac) were used to assess the level of similarity between the detected microbial communities. The beta diversity of the fecal microbiota was resultantly shown to be significantly reduced in the CKD compared with the HC group (qualitative ANOSIM, *R* = 0.253, *P* = 0.001) (Figure 1C). Similarly, the fecal microbiota of the patients with CKD and the HC subjects was clearly separated via the PCoA (Figure 1D). Although a few samples showed some overlap, the CKD and HC groups were obviously separated along PCoA2 and PCoA1, which explained 14.13 and 16.6% of the total variations, respectively. Together, these data suggest that the fecal microbial structure was significantly altered in patients with CKD compared to that in HC subjects in condition of the presence of OTU.

### Alteration of Taxa in the CKD and HC Groups

**Figure 2:**
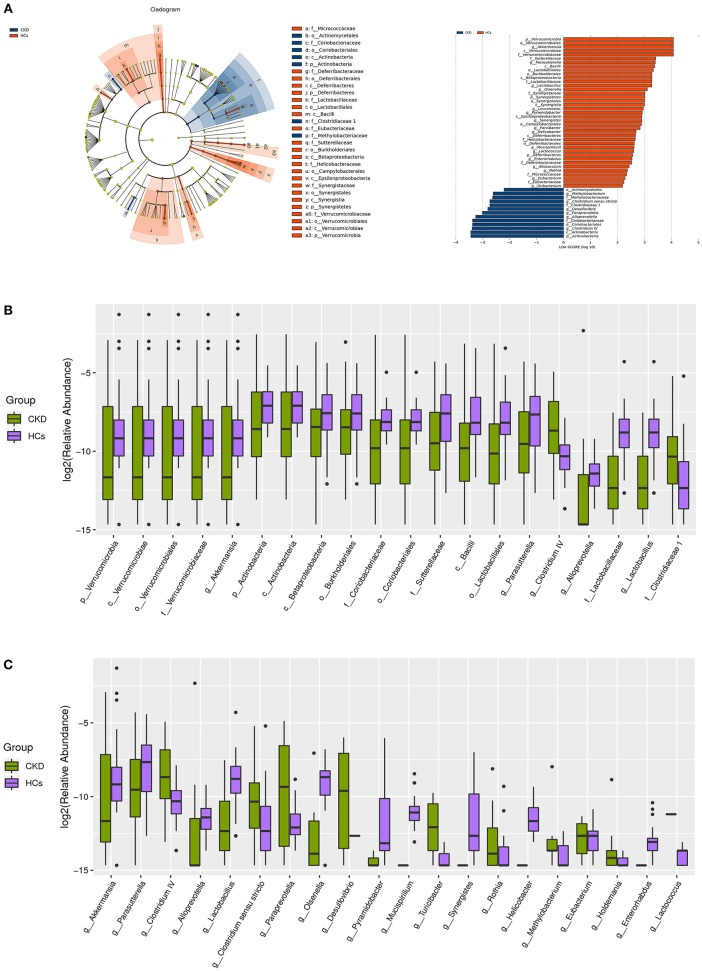
Taxonomic differences in the fecal microbiota exhibited by patients with chronic kidney disease (CKD) and healthy control (HC) subjects. **(A)** A Linear discriminant analysis (LDA; (log10) > 2) and effect size (LEfSe) analysis revealed significant differences (*P* < 0.05) in the fecal microbiota exhibited by the CKD (blue, negative score) and HC (red, positive score) groups. These analyses revealed the most differentially abundant taxa at the level of bacterial **(B)** phylum (p), class (c), order (o), family (f), and **(C)** genus (g) between the CKD (green) and HC (purple) groups.

A taxon-dependent analysis was conducted (using the RDP classifier software) to describe the composition of the CKD-associated fecal microbiota. The five bacterial phyla known to inhabit the human gut, comprising Firmicutes, Bacteroidetes, Actinobacteria, Proteobacteria, and Verrucomicrobia were identified in the collected samples. Two of these, Actinobacteria and Verrucomicrobia were found to be differentially abundant in the patients with CKD and the HC subjects. At the phylum level, the predominant sequences in the HC group were shown to comprise those of Firmicutes (41.52%), Bacteroidetes (37.69%), Proteobacteria (14.22%), Verrucomicrobia (3.09%), and Fusobacteria (2.00%) bacteria. Conversely, the most abundant sequences identified in samples from the patients with CKD were found to belong to Firmicutes (42.27%), Bacteroidetes (37.85%), Proteobacteria (16.70%), Actinobacteria (1.48%), and Verrucomicrobia (0.67%) bacteria. Notably, the relative abundance of the Verrucomicrobia and Actinobacteria phyla was higher (mean 3.09 vs. 0.67%, *P* = 0.001) and lower (mean 1.14 vs. 1.47%, *P* = 0.036) in the HC than in the CKD group, respectively (Figure 2B). Levels of other detected bacteria are shown in Figure 2B.

At the genus level, the bacteria detected in the HC groups were found to predominantly belong to the *Bacteroides* (28.95%), *Escherichia/Shigella* (11.75%), *Clostridium* XlVa (7.36%), *Alistipes* (4.96%), and *Veillonella* (4.61%) genera. Likewise, the most abundant genus in the CKD group was *Bacteroides* (32.88%), followed by *Escherichia/Shigella* (11.04%), *Phasco/arctobacterium* (4.76%), *Roseburia* (4.26%), and *Clostridium* XlVa (4.22%). Interestingly, the genera that were most significantly enriched in fecal samples from the HC compared to the CKD group were *Akkermansia* (mean 3.08 vs. 0.67%, *P* = 0.001) and *Parasutterella* (mean 0.93 vs. 0.47%, *P* = 0.007). Conversely, the *Lactobacillus* (mean 0.71 vs. 0.47%, *P* < 0.001), *Clostridium IV* (mean 0.59 vs. 0.11%, *P* = 0.015), *Alloprevotella* (mean 0.41 vs. 0.04%, *P* < 0.001), *Paraprevotella* (mean 0.25 vs. 0.04%, *P* = 0.001), *Clostridium sensu stricto* (mean 0.20 vs. 0.15%, *P* = 0.014), and *Desulfovibrio* (mean 0.12 vs. 0.00%, *P* = 0.025) genera were significantly enriched in fecal samples from the CKD compared to the HC group (Figure 2C).

A supervised comparison of the microbiota between the CKD and HC group was conducted via Linear discriminant (LDA) and effect size (LEfSe) analyses (since these are often used to identify the presence and impact of region-specific OTUs in different groups). A logarithmic LDA score cutoff of 2.0 was set to identify important taxonomic differences between the CKD and HC groups. The results of these analyses revealed remarkable differences in the fecal microbiota that were collected from the patients with CKD and the HC subjects. In particular, the relative abundances of the genera *Clostridium IV, Alloprevotella, Paraprevotella, Desulfovibrio*, and *Clostridium sensu stricto* were found to be higher in the patients with CKD than in the HC subjects (LDA score (log10) > 2), while both *Akkermansia* and *Parasutterella* were found to be enriched in the HC compared to the CKD group (Figure 2A).

### CKD-Associated Gut Biomarkers

**Figure 3:**
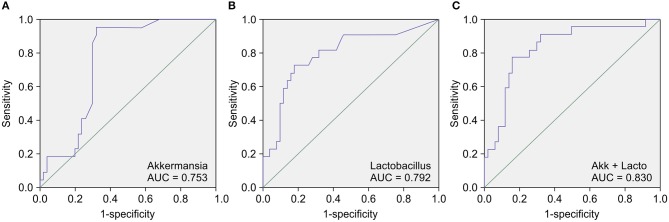
Receiver operating characteristic curve (ROC) analysis of the sensitivity and specificity of the differentially abundant genera as diagnostic factors for chronic kidney disease (CKD). The conducted analysis suggests that the relative abundances of **(A)** *Akkermansia*, **(B)** *Lactobacillus*, and most particularly, **(C)** of both *Akkermansia* and *Lactobacillus*, are likely promising diagnostic factors to distinguish between patients with CKD and healthy control (HC) subjects.

Next, we conducted a receiver operating characteristic (ROC) curve analysis to evaluate the value of the calculated relative abundances of various genera as CKD diagnostic factors. The area under the ROC curve (AUC) showed that the genera that were most closely correlated with CKD were *Akkermansia* (AUC = 0.753) (Figure 3A), *Lactobacillus* (AUC = 0.792) (Figure 3B), *Parasutterella* (AUC = 0.702), and *Clostridium IV* (AUC = 0.680). Notably, analyzing the combined diagnostic value of *Akkermansia* and *Lactobacillus* generated an AUC value of 0.830 (Figure 3C), which suggests that *Akkermansia* and *Lactobacillus* are likely promising potential diagnostic biomarkers for CKD.

### Potential Correlations Between the Fecal Microbiota and CKD Clinical Characteristics

**Figure 4:**
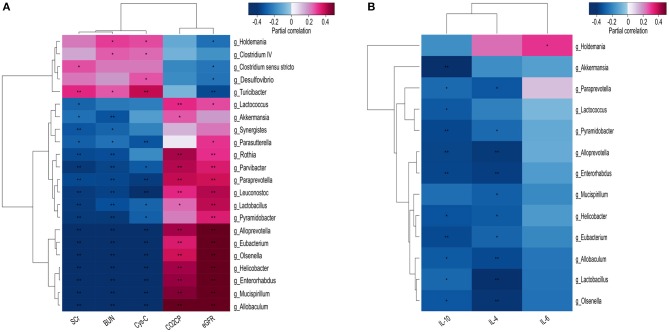
Heatmaps showing correlations between differentially abundant microbiota genera and chronic kidney disease (CKD) clinical parameters. Correlations were demonstrated between various differentially abundant fecal microbiota genera and **(A)** the specified CKD clinical characteristics, and **(B)** the production of inflammatory cytokines. BUN, blood urea nitrogen; CO_2_CP, carbon dioxide combining power; CysC, Cystatin C; eGFR, estimated glomerular filtration rate; IL, Interleukin; SCr, Serum creatinine. Spearman test, *P < 0.05, **P < 0.01.

We confirmed that CKD-specific changes (at the genus level) to the fecal microbiota were correlated with known CKD clinical parameters, including BUN, CysC, and SCr levels, as well as the eGFR and CO_2_CP. Specifically, most of the differentially abundant genera identified in the fecal microbiota, including *Parasutterella, Rothia, Lactobacillus, Olsenella, Paraprevotella, Lactococcus*, and *Helicobacter* were positively correlated with CKD severity indicators; however, a few others, including *Turicibacter, Clostridium sensu stricto, Desulfovibrio*, and *Holdemania* were negatively correlated with the eGFR (Figure 4A).

Since gut microbiota has been shown to mediate systemic chronic inflammation in various diseases, we next analyzed potential correlations between the differentially abundant fecal microbiota and the inflammatory factors IL-4, IL-6, and IL-10. *Akkermansia* was found to be significantly negatively correlated with IL-10 (*P* < 0.000), while nine other genera, comprising *Allobaculum, Alloprevotella, Enterorhabdus, Eubacterium, Helicobacter, Lactobacillus, Olsenella, Paraprevotella*, and *Pyramidobacter* were shown to be negatively correlated with IL-10 and IL-4 (*P* < 0.05). Only *Holdemania* was found to be positively correlated with IL-6 (*P* < 0.05) (Figure 4B).

### Predictive Function Analysis

**Figure 5:**
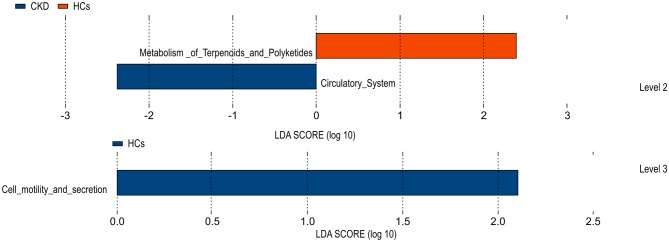
Functional predictions for the fecal microbiome exhibited by the patients with chronic kidney disease (CKD) and the healthy control (HC) subjects.

A Phylogenetic Investigation of Communities by Reconstruction of Unobserved States (PICRUSt) test, based on the constructed OTUs, was used to predict the function of the various gene sequences identified in the analyzed fecal microbiota, according to the Kyoto Encyclopedia of Genes and Genomes (KEGG) orthology database. The results of this analysis predicted that the fecal microbiome of the HC group was enriched for microbial gene function involved in terpenoid and polyketide metabolism, and cell motility and secretion. Conversely, that of the CKD group was predicted to be enriched for microbial genes associated with the circulatory system function. By comparing clustered orthologous gene sequences with the Kyoto Encyclopedia of Genes and Genomes (KEGG) orthology database, the microbial genes identified in the gut microbiota of the CKD and HC groups were predicted to be associated with polyketide metabolism and/or cell motility and secretion, and with circulatory system function, respectively (Figure 5). P values were calculated based on White's non-parametric *t*-test, and the Benjamini-Hochberg false discovery rate correction.

## Discussion

CKD is a prevalent, life-threatening disease that constitutes a major public health problem worldwide, for which few therapeutic options are currently available. The most frequent cause of death in patients with CKD is CVD complications, and the increased cardiovascular mortality risk exhibited by patients with CKD has been attributed to both traditional (e.g., hypertension, diabetes, and dyslipidemia) and non-conventional risk factors. In recent years, chronic systemic inflammation, often resulting from gut microbiota dysbiosis, has been identified as a major novel risk factor for CVD complications in CKD (Ruiz-Andres et al., 2016; Al Khodor and Shatat, 2017).

Several factors have been shown to alter the gut microflora in patients with CKD. For example, an increased concentration of urea in intracellular and extracellular fluids can lead to a massive urea influx (via passive diffusion) into the gastrointestinal tract, leading to the incorporation of urea into gut glandular secretions. This urea is decomposed to ammonia by urease produced by intestinal bacteria, leading to luminal pH changes that damage intestinal epithelial cells (Sabatino et al., 2015). Moreover, uric acid, which is the end product of purine metabolism in humans, is normally processed by the kidneys and excreted in the urine; however, in CKD, it is instead excreted by the colon, along with oxalate (Nallu et al., 2017). In addition, patients with CKD require a diet that includes only a severely limited amount of fruits, vegetables, and high-fiber products that are rich in potassium and oxalate. Diet has been shown to critically mediate the gut microbial flora, and in fact, these products contain indigestible dietary complex carbohydrates that normally comprise a primary nutrient source for gut microbiota. When carbohydrate availability is reduced, proteins are increasingly fermented by proteolytic bacteria such as *Clostridium* and *Bacteroides* to produce energy (via deamination); however, protein fermentation also produces potentially toxic metabolites (e.g., ammonia, phenols, and indoles) (Gryp et al., 2017). Finally, patients with CKD exhibit frequent arteriovenous access and other infections, and thus, require antibiotics. They similarly often require large quantities of phosphate-binding agents. Both drug types are well-known to modify the composition and/or structure of the gut microbiota (Huang et al., 2017).

Recent studies have linked gut microbiota dysbiosis to renal diseases, as evidenced by observed changes to the complexity and/or structure of the intestinal microbiota before and after renal transplantation (Lee et al., 2014) or kidney-stone formation (Ticinesi et al., 2018). Consistent with previous findings, our study confirmed that gut microbiota dysbiosis occurs in Chinese patients with CKD and, furthermore, identified differentially expressed bacterial taxa in patients with CKD. Indeed, the strength of the present study lies in its comprehensive description of microbial communities associated with CKD, and in particular, with CKD clinical characteristics, and the production of inflammatory factors. Moreover, the results of the conducted analyses showed that the diversity, overall richness, and structure (as indicated by the calculated alpha- and beta-diversity indices) of the gut microbiota are significantly reduced in Chinese patients with CKD compared to the HC subjects. This finding provides powerful evidence that the gut microbiota is clinically altered in CKD.

As discussed, five bacterial phyla are known to be present in the human gut, namely, Firmicutes, Bacteroidetes, Actinobacteria, Proteobacteria, and Verrucomicrobia. Of these, Bacteroidetes (*Bacteroides, Prevotella*, and *Xylanibacter*) and Firmicutes (*Ruminococcus, Clostridium, Lactobacillus, Eubacterium, Faecalibacterium*, and *Roseburia*) are normally the most predominant (Mahmoodpoor et al., 2017). The results of the present study showed that the most abundant phyla in both the CKD and HC groups were Firmicutes and Bacteroidetes, and indeed, their abundance was unchanged; however, the relative abundances of Verrucomicrobia and Actinobacteria were lower and higher in the CKD than in the HC groups, respectively. Notably, Actinobacteria are gram-positive bacteria that constitute one of the five largest bacterial phyla and produce antibiotic, anticancer, anthelmintic, and antifungal compounds that are important in biotechnology, medicine, and/or agriculture (Barka et al., 2015); however, the roles of both Actinobacteria and Verrucomicrobia in CKD are not yet clear.

Our study focused on genus-level changes to the gut microbiota, and thus, identified CKD-specific differences in the proportion of several bacterial taxa in the collected fecal samples. The most abundant genus observed in CKD patients was *Bacteroides*. This finding is in contrast to that of a previous study by Jiang et al. (2017), who found that the genus *Bacteroides* was enriched in ESRD patients. Further, in the study by Jiang et al. (2017), the genera *Escherichia/Shigella* were enriched in ESRD patients, and *Roseburia* was more abundant in controls, which is inconsistent with our results. In addition, the specific bacterial species of the indicated genera were different from those observed in our study. This may be due to differences in the study region and technical deviations, among others. On the other hand, Jiang et al. (2017) findings supported that bacteria producing SCFAs, especially butyrate were decreased in ESRD patients. Our results highlight the correlation between differentially abundant bacteria and inflammatory factors, and the probiotic strain *Akkermansia* decreased significantly in CKD patients.

In particular, the abundance of the known probiotic strain *Akkermansia* was obviously reduced, comprising 3.08% and only 0.67% of the microbiota in the HC subjects and patients with CKD, respectively. Conversely, the abundance of the probiotic *Lactobacillus* strain was slightly increased in the CKD compared to the HC group, and the abundance of several other bacterial genera, including *Parasutterella, Paraprevotella, Clostridium IV*, and *Alloprevotella*, also differed between the patients with CKD and the HC subjects; however, these changes were less significant than those observed for *Akkermansia*. Notably, *Parasutterella* has been previously shown to be associated with the pathogenesis of, and with chronic intestinal inflammation in patients with irritable bowel syndrome (IBS) (Chen et al., 2018).

*Akkermansia* or *Akkermansia muciniphila* is the only intestinal isolate of the highly branched Verrucomicrobia phylum. In healthy individuals, *Akkermansia* has been shown to comprise 1–4% of the total fecal microbiota from early life, consistent with our finding that the HC subjects in the present study exhibited a 3.08% abundance of *A*. *muciniphila*. Importantly, gut levels of *A. muciniphila* have been suggested to be negatively correlated with several diseases, including appendicitis, obesity, and diabetes (Ottman et al., 2017a), consistent with the negative correlation between *A. muciniphila* and severe-CKD indicators demonstrated herein.

*A. muciniphila* is an important mucus-degrading intestinal bacterium that encodes mucin-degrading enzymes that function to protect the gut barrier by increasing the thickness of the gut mucus. Moreover, mucin degradation leads to the production of carbon, energy, and nitrogen that support butyrate-producing bacteria (which cannot degrade mucins), which in turn provide energy (as butyrate) for colonocytes. Thus, *A. muciniphila* can be considered a key species of the mucosal layer; however, it also critically mediates host physiological functions by producing SCFAs, which are well-established to mediate host metabolism and inflammation, during mucin degradation (Hänninen et al., 2018). Finally, *A. muciniphila* has been demonstrated to help detoxify hydrogen sulfide. The present study found that the abundance of *A. muciniphila* was significantly decreased in patients with CKD compared to the HC subjects. While this finding is consistent with the hypothesis that *A. muciniphila* is a probiotic bacterium, further study is needed to evaluate whether reduced *A. muciniphila* levels aggravate CKD progression and/or promote CKD-associated CVD.

As discussed, gut microbiota dysbiosis has been shown to be associated with disease progression and CVD in CKD (Sabatino et al., 2017). Importantly, this study demonstrated a clear correlation between the presence of various microbiome genera and well-known clinical indicators of CKD progression, including BUN, SCr, and CysC levels; the CO_2_CP; and particularly the eGFR (which is known to be a sensitive indicator of renal function in patients with CKD), suggesting that the microbiota may mediate CKD pathogenesis. Our findings showed that BUN and SCr levels and eGFR were significantly correlated with the differential abundance of bacteria in individuals with, compared to without CKD. Specifically, the abundance of *Lactobacillus, Olsenella, Helicobacter*, and *Paraprevotella* was positively correlated with eGFR, and negatively correlated with BUN and SCr levels.

Chronic systemic inflammation has been widely investigated in recent years and shown to be an important contributing factor underlying CKD progression, including the incidence of CKD-associated CVD (Poesen et al., 2016). Accordingly, the levels of multiple pro- and anti-inflammatory cytokines, including IL-4, IL-6, IL-8, IL-10, and INF-γ, have been reported in CKD and suggested to contribute to CKD progression and CVD complications (Missailidis et al., 2016). IL-10 levels were associated with the reduction of kidney function and the risk of cardiovascular events (Yilmaz et al., 2014). However, IL-10 has been suggested to be an anti-inflammatory cytokine by some studies (Arenas-Padilla et al., 2018). Consistent with this, the present study showed that levels of the pro-inflammatory cytokines IL-4 and IL-6 significantly increased in the CKD compared to the HC groups. Moreover, the conducted correlation analysis of the differential abundance of various intestinal genera with these inflammatory factors showed that many bacteria were significantly negatively correlated with IL-4 and/or IL-10 levels, including those of the probiotic *Akkermansia* (IL-10 only) and *Lactobacillus* (IL-4 and IL-10) genera. Notably, Yoshifuji et al. (2016) previously found that *Lactobacillus* slowed the progression of kidney disease by improving the intestinal environment. Similarly, *Akkermansia* bacteria are currently undergoing intensive study as promising new generation probiotics. While its role in disease and pathogenesis require further investigation, *A. muciniphila* has been shown to lower serum endotoxin levels and islet toll-like receptor expression to promote regulatory immunity and to delay the development of diabetes (Greer et al., 2016). Other studies have moreover shown that *Akkermansia* bacteria are capable of improving enteritis and liver damage in mice (Ottman et al., 2017b; Seregin et al., 2017; Wu et al., 2017). Therefore, we propose that *Akkermansia* bacteria may improve renal function in CKD patients and alleviate the chronic systemic inflammation associated with CKD. However, this requires further in-depth study. The results of the present study revealed that the patients with CKD exhibited a reduced abundance of intestinal probiotics and elevated levels of inflammatory factors, and furthermore, that levels of these probiotics and inflammatory factors were negatively correlated. Nevertheless, little is known about the molecular host-microbiome interactions that influence disease progression and chronic systemic inflammation in CKD. Previous studies have indicated that *Bifidobacterium* and *Lactobacillus* can play a therapeutic role by up-regulating the expression of IL-10 in serum (Wu et al., 2017; Arenas-Padilla et al., 2018). Our study found that *Akkermansia* was negatively correlated with IL-10 level; therefore, the role of *Akkermansia* and IL-10 in CKD needs further in-depth study.

The temporary stability of the gut microbiota has important implications for use of the microbiota as a diagnostic tool. Prognostic factors (e.g., SCr levels and the eGFR) that predict outcomes in CKD have been widely evaluated; however, to date, the value of the gut microbiota as a prognostic biomarker has not been assessed. The present study revealed significant differences in the abundance of *Akkermansia* and *Lactobacillus* bacteria in Chinese patients with CKD and HC subjects, suggesting that *Akkermansia* and/or *Lactobacillus* levels in the fecal microbiota may be promising diagnostic or prognostic markers for CKD (*Akkermansia*, AUC = 0.753; *Lactobacillus*, AUC = 0.792, combined, AUC = 0.830). Furthermore, the conducted functional predictions suggested that these genera likely mediate (or protect against) CKD pathogenesis by modulating terpenoid and/or polyketide metabolism.

In conclusion, the results of the present study indicate that gut microbiome components are significantly different in HC subjects and patients with CKD and that the differential abundance of some genera is correlated with CKD clinical characteristics and the production of inflammatory factors. Thus, we suggest that these changes to the gut microbiota may be promising diagnostic markers for CKD. In particular, we showed that the abundance of the probiotic *Akkermansia* genus was significantly decreased in the intestinal microbiota of patients with CKD and was significantly negatively correlated with the production of the inflammatory factor IL-10. Further research is needed to elucidate the mechanism(s) by which *Akkermansia* and IL-10 interact to mediate CKD disease progression. Nevertheless, the data presented herein support that novel therapeutic strategies to modify the gut microbiota composition in CKD may have the potential to mitigate chronic systemic inflammation, and thereby improve outcomes for patients with this disease.

## Data Availability

All relevant data is contained within the manuscript. The raw data supporting the conclusions of this manuscript will be made available by the authors.

## Author Contributions

FL: clinical analyses and manuscript writing. MW: sample collection and DNA extraction. YZ: sequencing. JW: sequencing analyses and statistical analyses. RL: study design, project supervision, and manuscript revision.

### Conflict of Interest Statement

The authors declare that the research was conducted in the absence of any commercial or financial relationships that could be construed as a potential conflict of interest.
